# Recent Acquisition of Functional m6A RNA Demethylase Domain in Orchid Ty3/Gypsy Elements

**DOI:** 10.3389/fpls.2022.939843

**Published:** 2022-07-04

**Authors:** Luis Alvarado-Marchena, Mireya Martínez-Pérez, Frederic Aparicio, Vicente Pallas, Florian Maumus

**Affiliations:** ^1^Instituto de Biología Molecular y Celular de Plantas (IBMCP), Consejo Superior de Investigaciones Científicas, Universitat Politècnica de València, Ingeniero Fausto Elio, Spain; ^2^INRAE, URGI, Université Paris-Saclay, Versailles, France

**Keywords:** epitranscriptomic, LTR retrotransposons, m6A RNA methylation, Phalaenopsis (orchids), plants, transposable elements

## Abstract

Long terminal repeats (LTR) retrotransposons are transposable elements (TEs) representing major components of most plant genomes. The fixation of additional conserved protein domains in their genomes is considered a rare event in the course of their evolution. Such changes can bring novel functions and increase their fitness by playing a role in the regulation of their replicative cycle or by affecting their integration landscape so that the detection of new domains can in turn reveal important aspects of host-TE interactions. We have mined angiosperm genomes for the presence of additional domains in LTR retrotransposons. We report a lineage of large (25 kbp) Gypsy-type elements in the genomes of Phalaenopsis orchids that contain an additional open reading frame containing a 2-ODD domain with close similarity to those responsible for m6A RNA demethylase activity in AlkB proteins. By performing *in vitro* assays, we demonstrate the RNA binding capability and the demethylase activity of the Gypsy-encoded AlkB protein, suggesting it could be functional against cognate TE mRNA or any cellular RNA *in planta*. In line with recent literature, we propose that the fixation of an RNA demethylase in this lineage of LTR retrotransposons may reflect an important role for epitranscriptomic control in host surveillance against TEs.

## Introduction

Transposable elements (TEs) are genetic sequences whose primary function is to mediate their own replication and integration in host genomes from all domains of life. Owing to their duplicative properties and their repetitive nature, TEs have plethora of impacts on eukaryotic genomes such as influencing their size (e.g., TEs contribute 45 and 80% of the human and maize genomes, respectively), mediating genomic rearrangements, generating genetic diversity, distributing regulatory sequences, shaping the architecture of chromosomes and chromatin, and contributing to the definition of sexual chromosomes (Kazazian, 2004). Two main classes are recognized: class I elements (also called retrotransposons) have a so-called copy-and-paste replication mechanism involving a genomic RNA (gRNA) intermediate, whereas class 2 elements have a cut-and-paste mechanism without gRNA intermediate (Wicker et al., 2007). While TE superfamilies share a conserved backbone of protein domains and key regulatory motifs in a specific organization, they have also evolved significant changes in their architecture along their evolutionary history. Variations can be observed for instance in protein domain content and striking examples of stable acquisitions of domains have been described at variable depth in the phylogeny of TE superfamilies (Llorens et al., 2009). Such major transitions along the evolution of TE superfamilies can have profound consequences on TE replication and regulation as well as on their interaction with host organisms.

Long terminal repeats retrotransposons are Class I TEs that present long terminal repeats (LTRs) and found throughout eukaryotes. They comprise three superfamilies: Bel/Pao, Ty1/Copia, and Ty3/Gypsy, the latter two being commonly abundant in plant genomes (Wicker et al., 2007). Both superfamilies contain the same assortment of core domains typically encoded by the gag and pol genes, i.e., the GAG, protease, integrase, reverse transcriptase (RT) and ribonuclease H domains. Several examples of protein domain acquisition were reported among LTR retrotransposons. For instance, specific lineages from both Copia- and Gypsy-type elements have acquired envelope-like (ENV) genes sharing similarity to those found in retroviruses (Laten et al., 1998; Wright and Voytas, 1998). As another example, one of the major branches of the Gypsy-type superfamily, the chromoviruses, contains a CHROMO domain that was shown to interact with histone marks and to target integration toward heterochromatin (Marin and Llorens, 2000; Gorinsek et al., 2004; Gao et al., 2008). Furthermore, giant Gypsy-type LTR retrotransposons from the planarian *Schmidtea mediterranea* were found to contain a whole collection of ORFs encoding domains generally absent from this TE superfamily (Arkhipova and Yushenova, 2019). In plants, several additional ORFs were detected across different lineages of Ty3/Gypsy elements although they most often lack known conserved protein domains (Steinbauerova et al., 2011; Neumann et al., 2019; Vicient and Casacuberta, 2020).

The activity of TEs is tightly controlled by host cell machinery at different levels of their replication cycle throughout plant development. For instance, TEs are known to be transcriptionally silenced through epigenetic regulation such as DNA methylation and post-transcriptionally inactivated through different small RNA pathways (Borges and Martienssen, 2015; Matzke et al., 2015).

In different eukaryotes, such as mammals, yeasts and plants, a large variety of RNAs can be the subject of post-transcriptional modifications. N6-methyladenosine (m6A) is one of the most common and abundant modification in RNA molecules present in eukaryotes and may be referred to as the “fifth base” of RNA. Several studies have shown that m6A participates in different aspects of RNA biology such as the regulation of mRNA stability (Wang et al., 2014), translation (Wang et al., 2015), and protein-RNA interactions (Liu et al., 2015) although recently evidence of a direct role in promoting translation has been questioned (Zaccara and Jaffrey, 2020). In contrast to mammals, studies on the function of m6A modification in plants are scarcer (Arribas-Hernandez and Brodersen, 2020). Transcriptome-wide profiles in *Arabidopsis thaliana* detected the m6A modification in over two-thirds of the mRNAs (Wan et al., 2015). m^6^A depletion was described to increase the expression of m^6^A-targeted mRNAs involved in plant developmental control (Shen et al., 2016) whereas Anderson et al. (2018) found that m6A stabilizes transcripts by inhibiting ribonucleolytic cleavage directly upstream of these modification sites in *Arabidopsis*. This RNA modification has been recently demonstrated to be important to regulate salt (Hu et al., 2021a; Zheng et al., 2021) and drought (He et al., 2021) stress tolerance, the infection cycle of a plant RNA virus (Martínez-Pérez et al., 2017), the stability of flowering-related genes (Duan et al., 2017), the expansion and ripening of tomato fruits (Hu et al., 2021b) or the yield and biomass of rice and potato plants (Yu et al., 2021). Thus, manipulation of plant RNA m6A methylation could be an encouraging strategy to improve plant growth and crop yield.

RNA m6A methylation is a reversible modification. In mammals, the protein complex known to add the methyl group to RNA (writers) includes the catalytic RNA methyltransferase METTL3, the allosteric activator METTL14, and several assisting factors such as WTAP. The demethylase enzymes (erasers) FTO and ALKBH5 have been shown to remove m6A from RNA (Hu et al., 2019). In plants, orthologs of METTL3 and METTL14 (MTA and MTB, respectively) and some of the assisting cofactors were characterized (Shen et al., 2016; Ruzicka et al., 2017). In fact, analysis of *Arabidopsis mta* knockdown mutants revealed that MTA is required for m6A mRNA methylation and essential for normal growth and development (Zhong et al., 2008; Yue et al., 2019). Furthermore, ALKBH5 orthologs found in *A. thaliana*, ALKBH9B and ALKBH10B, were shown to have RNA demethylation activity (Duan et al., 2017; Martínez-Pérez et al., 2017). Interestingly, while *alkbh9b* knockout mutants do not show differences in overall plant RNA m6A methylation level, its depletion results in hypermethylation of alfalfa mosaic virus (AMV) RNA and impairment of systemic infection (Martínez-Pérez et al., 2017, 2021). In addition, several studies have pointed out the varying roles of m6A modifications in the life cycle of animal- and plant-infecting RNA viruses (Gonzales-van Horn and Sarnow, 2017; Williams et al., 2019; Yue et al., 2022). More recently, RNA m6A methylation was also reported to play a role in the control of the replication of mammalian endogenous viruses and retrotransposons elements in contrasting ways. Indeed, mRNAs derived from endogenous retroviruses are methylated and the levels of m6A RNA methylation negatively affect their accumulation in mouse embryonic stem cells (ESCs) (Chelmicki et al., 2021). On the other hand, mRNAs of young LINE-1 retrotransposons are strongly methylated but positively promote LINE-1 retrotransposition in mouse ESCs (Xiong et al., 2021). While the control of TEs by balancing RNA m6A methylation levels appears as an emergent, boiling field of investigation, there is no clue at this stage how this modification may affect different types of TEs and whether it also plays a role in plants.

In this study, we explore the protein domain content of LTR retrotransposons across angiosperm genomes and found several previously unreported domains in Gypsy-type elements. Most intriguingly, we discovered a family of Gypsy-type elements in orchids that contain a functional RNA m6A demethylase acquired from the AlkB protein family.

## Materials and Methods

### Identification of Protein Domains

We first searched for reverse transcriptase in 121 plant genome assemblies (Supplementary Table 1) representing about 90 Gb in total). To this end, we first prepared a query library by clustering the 165 RT amino acid sequences of Gypsy and Copia reference elements taken from Gypsy database version 2 (Llorens et al., 2011) at a depth of 40% identity and we selected one representative sequence per cluster (*n* = 35). We then used the resulting RT library to search for homologous regions in target genomes with tBLASTn from ncbi blast + package. All overlapping hits on genomes were merged and the corresponding FASTA sequences within the expected size range (520–840 bp) were extracted (*n* = 1,949,052). To avoid sparing unnecessary computational time, during following steps, RT sequences from each species were clustered at a threshold of 95% identity using MMseqs2 (Steinegger and Soding, 2017) and a maximum of 50 sequences per group were selected for downward analysis. The genomic positions of RT coding regions were extended to 5 kb upstream and downstream and the corresponding sequences were extracted (*n* = 1,456,640). Extended hits were then clustered using mmseqs2 (with parameters -c 0.5 –max-seq-len 15000), and the groups containing at least 5 sequences were aligned with MAFFT (Katoh et al., 2002). A consensus sequence was then generated for each sequence alignment through the modules “msa2profile” (with parameters –match-mode 1 –match-ratio 0.5) and “profile2consensus,” resulting in 25,565 consensus sequences. Each consensus was compared to the CDD database (Marchler-Bauer et al., 2010) using MMseqs2. The classes of domains that are commonly found across LTR retrotransposons were filtered (belonging to Gag, protease, reverse transcriptase, ribonuclease, integrase, and Chromo categories) to obtain the names of uncommon conserved protein domains detected in at least 5 consensuses established from a same species (i.e., corresponding to a TE family). To address the fraction of the consensus sequences representing LTR retrotransposons, we compared each one to a library of reference aa RT sequences from Copia, Gypsy, DIRS, endogenous retroviruses, Caulimoviridae, and LINEs using BLASTx. The consensuses corresponding to LTR retrotransposons were identified from their best hit (highest bit score) against RT from Copia or Gypsy. Intrinsically disordered regions (IDRs) within Gypsy_Pa_2799 ORF2 were predicted using PrDOS with default settings (Ishida and Kinoshita, 2007).

### Phylogenetic Analysis

To build the reverse transcriptase tree, we extracted the RT domain from Gypsy_Pa_2799 ORF1 and combined it with those from 96 reference elements obtained from the Gypsy database (Llorens et al., 2011) to produce a multiple sequence alignment using MAFFT (Katoh et al., 2002) with the option “–maxiterate 1000.” The resulting alignment was submitted to IQ-TREE (Minh et al., 2020) for model testing (best-fit model: LG + R6 chosen according to BIC) and phylogenetic analysis with 100 bootstrap replicates. For the AlkB tree, we first retrieved the sequences from eight predicted human AlkB proteins (Kurowski et al., 2003). The human proteins were used as queries to identify and collect homologs in *A. thaliana.* We then combined the 2-ODD domain from Gypsy_Pa_2799 ORF2 to these references sequences to produce a multiple sequence alignment using MAFFT with the options “–maxiterate 1000 –dash –originalseqonly.” The resulting alignment was trimmed manually at the extremities to keep only the 2-ODD domain and then submitted to IQ-TREE for model testing (best-fit model: LG + R3 chosen according to BIC) and phylogenetic analysis with 100 bootstrap replicates.

### Characterization of Gypsy-Type Elements Encoding the 2-ODD Domain

LTRharvest (Ellinghaus et al., 2008) was launched on *P. equestris* and *P. aphrodite* genomes using the options “-maxdistltr 20000 -maxlenltr 3000 -mindistltr 5000.” For each genome, the LTRharvest predictions were clustered using MMseqs2 (with parameter -c 0.5) (Steinegger and Soding, 2017) and the clusters containing at least five sequences were aligned with MAFFT (Katoh et al., 2002). A consensus sequence was then generated for each sequence alignment through the modules “msa2profile” (with parameters –match-mode 1 –match-ratio 0.5) and “profile2consensus.” Each consensus was compared to the CDD database (Marchler-Bauer et al., 2010) using MMseqs2 and those containing the 2-ODD domain (pfam13532) were selected (*n* = 15) and aligned using MAFFT. The *P. aphrodite* genome coverage was obtained by using these 15 consensus sequences as a library with RepeatMasker where accepted divergence between target and query was limited to 20% (−div 20) tRNA.

### Purification of ALKB Proteins

The open reading frame of the putative Gypsy_Pa_2799 protein and the atALKBH9B gene as well as the paALKBH5-like His448Ala-mutant were chemically synthesized for subsequent periplasmic production strategy (signal peptide present in the N-terminal of the proteins) and cloned into pET28 plasmid fused to a Histidine tag at its C-terminal (ProteoGenix, Schiltigheim, France). Proteins were expressed in BL21 *Escherichia coli* cells and purified by immobilized metal affinity chromatography (IMAC) in native conditions following manufacturer recommendations.

### Electrophoretic Mobility Shift Assays

Nucleic acid-protein interactions were analyzed by electrophoretic mobility shift assay (EMSA). For these assays 5 ng of DIG-labeled riboprobe of 5′UTR of AMV RNA 3 transcripts was denatured (5 min at 85°C) and cooled at room temperature for 15 min. Different amounts of purified His:paALKBH5-like fusion protein (0–360 ng) were added and incubated for 30 min at 4°C in 10 μL of binding buffer (10 mM Tris–HCl pH 8.0, 100 mM NaCl and 50% glycerol), and 1U of RiboLock RNase inhibitor (Thermo Fisher Scientific). The samples were then resolved in 1.5% TAE agarose gels, capillary transferred to positively charged nylon membranes (Hybond-N, GE Healthcare, Amersham) and fixed by UV. Finally, localization of the DIG-labeled probes was achieved by development with an anti-DIG system (Roche Diagnostics) according to the manufacturer’s protocol. Northwestern assays were carried out as previously described (Pallas et al., 1999). Briefly, purified proteins (GST, His:paALKBH5-like or its mutant variant H448A) were electrophoresed in 12% SDS/PAGE and transferred to nitrocellulose membranes. Membranes were incubated overnight at 4°C in Renaturing Buffer (10 mM Tris–HCl pH 7.5, 1 mM EDTA, 0.1 M NaCl, 0.05% Triton X-100, 1X Blocking Reagent, Roche). After this, membranes were incubated with 20 mL of the same buffer containing 50 ng/mL of the AMV sgRNA 4 labeled with digoxigenin for 3 h at 25°C.

### *In vitro* Demethylation Assays

The m6A demethylase activity assay was performed by incubating 0.75 μg of m6A monomethylated ssRNA (Dharmacon, Inc., 5′-AUU GUC A[m6A]C AGC AGC-3′) and 2.5 μg of protein for 3 h at 37°C in a reaction mixture containing 50 mM HEPES buffer (pH 7.0), 10 μM α-ketoglutarate, 100 μM L-ascorbic acid ascorbate and 20 μM (NH_4_)2Fe(SO_4_)2⋅6H_2_O. Reactions were quenched by heating them at 95°C for 10 min. Digestion of nucleotides to nucleosides and the m6A/Adenosine (m6A/A) quantification by ultra-performance liquid chromatography–photodiode detector–quadrupole/time-of-flight–mass spectrometry (UPLC-PDA-TOF-MS) analysis of ssRNA oligonucleotide and total RNA were performed as described by Martínez-Pérez et al. (2017). Proteins used in this assay were the RNA-demethylase atALKBH9B (Martínez-Pérez et al., 2017) as positive control, the *P. aphrodite* Gypsy-element that codes a hsALKBH5-like (paALKBH5-like) and the paALKBH5-like His448Ala-mutant. This mutation affects the putative motif involved in Fe (II)-binding motif/iron core (H448xD…H), characteristic of the 2OG-FeII-dependent dioxygenase superfamily and the AlkB family (Lu et al., 2014). Three independent replicas were made with each of the proteins under study.

## Results

### Screen for Non-canonical Domains

**FIGURE 1:**
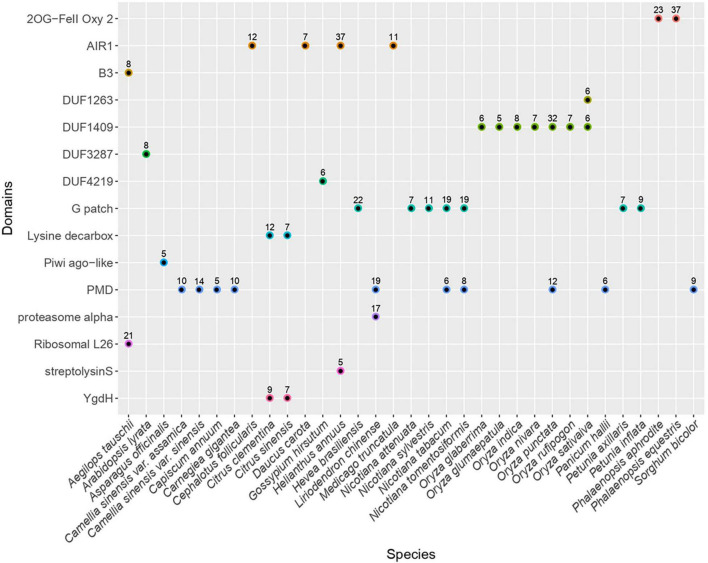
Distribution of frequent extra conserved protein domains across LTR retrotransposons. Plot representing the distribution of the different types of non-canonical conserved protein domains across plant species. Only the domains found in at least five consensus sequences from a given species (passing the n5 filter) are shown. The numbers above each dot indicate the number of consensuses in which each domain was detected in the respective species. The canonical domains found in LTR retrotransposons as well as the hitherto well described non-canonical domains (including chromo and ATHILA domains) are not shown. The redundancy associated to hits against homologous profiles from the CDD database has been collapsed to keep only one representative domain per domain family. The full chart of non-canonical domains detected is available in Supplementary Figure 1.

We searched for non-canonical protein domains embedded in LTR retrotransposons across 121 angiosperm genomes (Supplementary Table 1). To this end, we have first established a generic methodology to build a library of consensus sequences representing LTR retrotransposons from each plant genome. In brief, we identified genomic regions containing LTR retrotransposon sequences using the reverse transcriptase domain as a marker. The regions sharing high sequence identity were clustered and the sequences from each cluster were aligned to infer a consensus sequence. In total, we generated 25,565 consensus sequences, including 4,811 and 20,288 corresponding to Copia- and Gypsy-type elements, respectively (Supplementary Data). In addition, 423 consensus sequences were classified as endogenous Caulimoviridae and five were classified as LINE-type non-LTR retrotransposons. We have next scanned each consensus sequence for the presence of conserved protein domains by searching for profiles from the CDD database (Marchler-Bauer et al., 2010). After filtering those domains typically found in LTR retrotransposons, including Chromo domain models found in Chromoviruses, we have extracted the list of putative supplementary domains that were detected (Figure 1). Here, we focused on domains found in at least five consensus sequences from a given species (hereafter referred to as the “n5” filter) to empirically select evolutionary stable fusion events and to avoid describing potentially less meaningful fusions (charted on Supplementary Figure 1).

Applying this method, we detected 15 domains that are not commonly found in LTR retrotransposons, all being detected in Gypsy-type elements as opposed to Copia-type. Among the plant genomes analyzed here, seven atypical domains were found across several species, the most frequent being PMD, G-patch and AIR1.

The PMD domain (Plant Mobile Domain, PF10536), which function is unknown was the most frequent as it was detected in consensuses from ten plant species belonging to monocotyledons and dicotyledons (40 species when skipping the n5 filter). Though not considered a canonical Gypsy-encoded domain, PMD was previously described to associate with plant Gypsy-type elements (Steinbauerova et al., 2011) and MULE DNA transposons (Babu et al., 2006). In *Arabidopsis*, at least two PMD-containing proteins (MAIN and MAIL1) play a role in transcriptional gene regulation and TE silencing (Ikeda et al., 2017; Nicolau et al., 2020).

The G-patch domain (PF01585), containing a glycine-rich motif, is found in several eukaryotic RNA-processing proteins and is hypothesized to mediate binding to RNA. Here, this domain was found in LTR-RT consensus sequences from seven species (16 species when skipping the n5 filter) distributed across different clades of dicotyledons including the Rosids *Hevea brasiliensis* (order Malpighiales), the Asterids *Chenopodium quinoa* (order Caryophyllales), and *Nicotiana sp.* and *Petunia sp.* (order Solanales). Interestingly, the G-patch domain is also found in type D retroviral polyproteins (Aravind and Koonin, 1999).

Similarly, the AIR1 domain (Arginine methyltransferase-interacting protein), which contains a Zn-finger motif, was found in four different species (22 species when skipping the n5 filter) including the Asterids *Daucus carota* (order Apiales) and *Helianthus annuus* (order Asterales) as well as the Rosids *Medicago truncatula* (order Fabales) and *Cephalotus follicularis* (order Oxalidales). The AIR1 domain overlaps with the DNA binding universal minicircle sequence binding protein (UMSBP) domain which was previously described in some giant Gypsy-type LTR retrotransposons (Arkhipova and Yushenova, 2019) as well as in some non-LTR retrotransposons (Saint-Leandre et al., 2019).

Besides these most frequent domains, others were found in consensuses derived from a narrower range of species. For instance, the DUF1409 domain (PF07197), Domain of Unknown Function, was found in consensus sequences derived from seven different species from the genus *Oryza*. The taxonomic distribution of this domain appears to be restricted to the Poaceae family. Likewise, in line with a previous report (Steinbauerova et al., 2011), the YgdH domain was detected in consensus sequences obtained from two species of the *Citrus* genus, *Citrus sinensis* and *Citrus clementina* as well as in *P. trichocarpa* when n5 filter is disabled. YgdH domain is otherwise found in LONELY GUY protein family whose members play a pivotal role in regulating cytokinin activity in *Arabidopsis* (Kuroha et al., 2009) and the cell cycle in the green alga *Chlorella variabilis* (Nayar, 2020).

In addition, Ribosomal protein L26 domain was found in consensus sequences from *Aegilops tauschii* only. It contains the KOW RNA-binding motif which is shared so far among several families of ribosomal proteins (Kyrpides et al., 1996). Still from the sole *A. tauschii* genome but in different consensuses, we detected the B3 domain, a DNA binding domain found in plant transcription factors with diverse functions in plant growth and development. We also identified LTR-RT consensus sequences in *Asparagus officinalis* that contain the second linker region (ArgoL2) found in PIWI- and Argonaute-like proteins. It contains residues that were described to contribute to direct the path of the phosphate backbone of nucleotides 7 through 10 in 20-nucleotides small RNAs (Faehnle et al., 2013).

Last but not least, we detected a protein domain with similarity to 2-oxoglutarate/Fe(II)-dependent dioxygenases (2-ODDs) superfamily (cl/PFAM13532) in 38 and 23 consensus sequences established from the genome of the orchids (family Orchidaceae) *Phalaenopsis equestris* and *Phalaenopsis aphrodite*, respectively (Supplementary Data). Such domain was not detected in the consensuses derived from any other plant genome selected for this screen (even without the n5 filter), including those from several other orchids (*Dendrobium catenatum*, *Dendrobium huoshanense*, *Gastrodia elata*, *Apostasia shenzhenica*, and *Vanilla planifolia*). 2-ODDs compose a large superfamily of non-heme iron-containing domains that catalyze highly diverse oxidative reactions including hydroxylation, demethylation and desaturations in bacteria, fungi, plants, and metazoa (Herr and Hausinger, 2018). In plants, 2-ODDs are involved in a wide range of biological processes, including DNA repair, RNA and histone demethylation, hormone biosynthesis and the production of secondary metabolites (Farrow and Facchini, 2014; Kawai et al., 2014; Martínez-Pérez et al., 2017).

### Phalaenopsis 2-ODD-Containing Elements Are Among Giants

**FIGURE 2:**
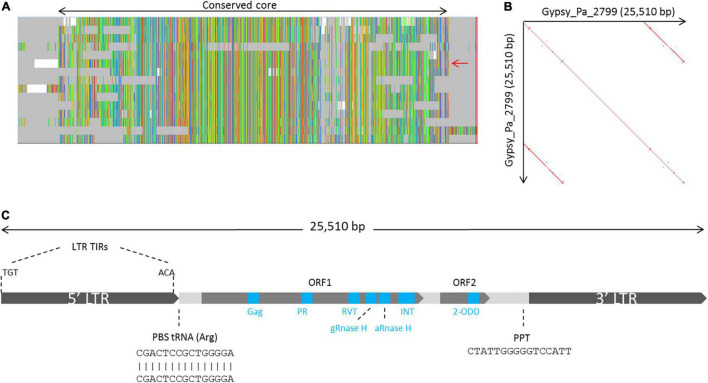
Organization of the family of Gypsy-type elements with 2-ODD domain in *P. aphrodite*. **(A)** Overview of the multiple sequence alignment of the 15 *P. aphrodite* consensus sequences showing their conservation and the main variations between them. The whole alignment spans 33,269 bp. The black, two-headed arrow, spans over the fraction of the alignment which is well conserved across most consensus sequences (representing 28,280 bp). The red arrow indicates the selected reference consensus for this family (Gypsy_Pa_2799). **(B)** Dot plot of the Gypsy_Pa_2799 consensus sequence highlighting its LTRs. **(C)** Schematic representation depicting the structure and main features of the Gypsy_Pa_2799 consensus sequence including the LTRs and their terminal inverted repeats (LTR TIRs), the primer binding site (PBS), the putative polypurine tract (PPT) as well as the two different ORFs and the protein domains they contain (blue boxes, not to scale).

We further investigated the structure and classification of the Phalaenopsis 2-ODD-containing elements. From our initial screen for LTR-RT elements across plant genomes, none of the 2-ODD-containing consensus sequence reconstructed from Phalaenopsis genomes presented long terminal repeats, indicating that those sequences might be truncated. This artifact is methodologically possible in case of large LTR-RT elements as our approach limits the reconstruction of consensus sequence to the regions 5 kb upstream and downstream of RT domains. We used LTRharvest software (Ellinghaus et al., 2008) to investigate the structure of full length corresponding elements. LTRharvest predictions were clustered on a similarity basis and the sequences from each cluster were aligned to derive consensus sequences. Following this approach, we obtained 254 and 178 consensuses from the *P. aphrodite* and *P. equestris* genome assemblies, respectively. After searching for conserved protein domains across these sequences, we identified 15 consensuses encoding the 2-ODD domain in *P. aphrodite* (Supplementary Data) but none in *P. equestris*, probably due to lesser contiguity level in this genome. The alignment of the 15 *P. aphrodite* consensuses revealed a core of high sequence conservation with some consensuses presenting structural variations (Figure 2A). We selected one sequence which covers the whole core alignment as representative (hereafter referred to as Gypsy_Pa_2799) to analyze its structural features. The Gypsy_Pa_2799 consensus sequence is 25,510 base pairs (bp) long and comprises 6,425 bp LTRs at the extremities (Figure 2B). The internal sequence encodes two open reading frames (ORFs): ORF1 (2640 aa) contains all the conserved protein domains typically found in Gypsy-type elements in a single polyprotein while ORF2 (565 aa) carries the 2-ODD domain at its C-terminus (Figure 2C). We then addressed the overall contribution of the 2-ODD containing elements to the *P. aphrodite* genome assembly. Using the fifteen selected consensus sequences derived from LTRharvest as input library (Figure 2A) for RepeatMasker annotation, we obtained approximately 88 Mb coverage, i.e., 8.6% of the genome assembly size (9.7% when excluding assembly gaps).

**FIGURE 3:**
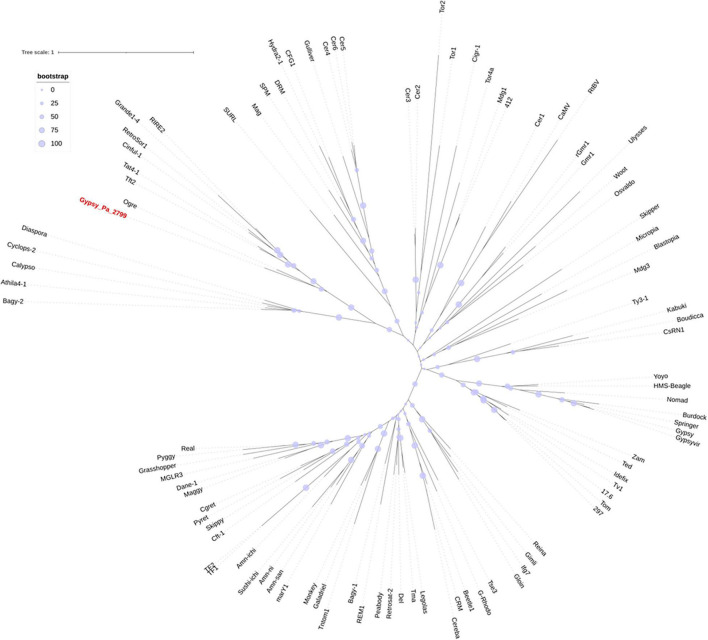
Phylogenetic tree of the Gypsy-encoded reverse transcriptase domain. The tree shows the phylogenetic relationships between reverse transcriptase domains from Gypsy_Pa_2799 (labeled in red) and reference elements from the Gypsy database. The bootstrap values are indicated as circles of size proportional to intervals of values described in the top left panel.

We next investigated the phylogenetic relationship of 2-ODD-containing elements with other Gypsy elements. After extracting the aa RT domains from Gypsy_Pa_2799, we found that they are highly conserved, showing above 90% identity with each other, so we selected a single RT sequence as representative. We aligned this sequence with RT sequences taken from a diversity of reference Gypsy-type elements obtained from the Gypsy database to address their evolutionary relationships. The phylogenetic tree indicates that RT domains from 2-ODD-containing elements are sister to the Ogre element from *Pisum sativum* (Neumann et al., 2003) and nested within the Tat family (Figure 3). This phylogenetic placement is in line with several features of the orchid elements. First, the relatively large size compared to other Gypsy-type elements, largely attributed to the size of LTRs, is typical of Ogre elements. Second, the primer binding site (PBS) is similar to that observed across Ogre elements, i.e., antisense to tRNA-Arginine. In addition, as reported across elements from the Tat family (Ustyantsev et al., 2015), the orchid elements also present a dual RNase H domain. However, important structural differences with Ogre elements can be observed as the Gag and Pol polyproteins are separated by a putative intron in the latter while they form a single ORF in Gypsy_Pa_2799 and as Ogre elements typically lack the additional ORF encoding the 2-ODD domain.

### Phalaenopsis 2-ODDs Are Homologous to Plant RNA Demethylases

**FIGURE 4:**
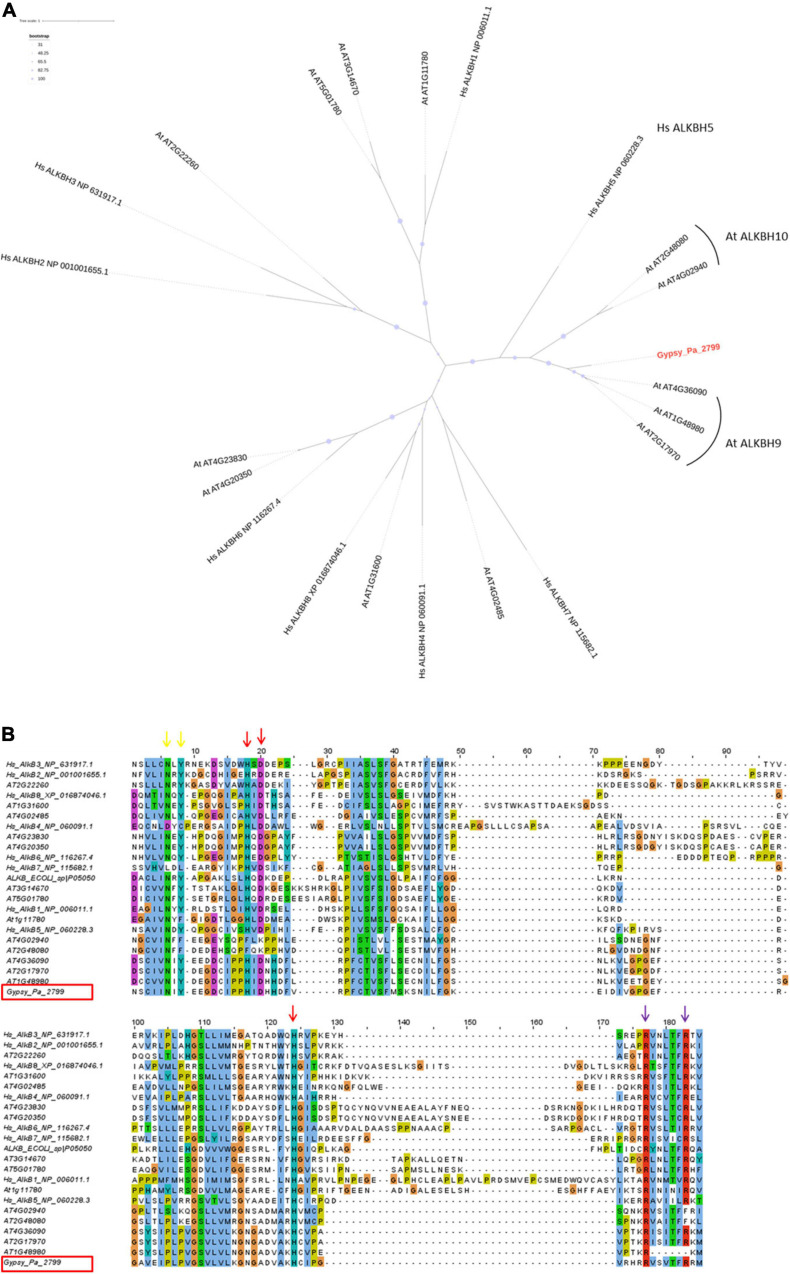
Characterization of the Gypsy-encoded 2-ODD domain. **(A)** Phylogenetic tree inferred from the alignment of Gypsy_Pa_2799 (labeled in red) 2-ODD domain with AlkB homologs from *E. coli*, *A. thaliana*, and *H. sapiens*. The bootstrap values are indicated as circles of size proportional with intervals of values described in the top left panel. **(B)** Conservation of co-factor binding residues in the 2-ODD domain across the alignment used in **(A)**. The alignment view is truncated to positions corresponding to residues 188–285 in human ALKBH5. The columns corresponding to the metal ion binding residues (ALKBH5 His-204, Asp-206, and His-266) are indicated by red arrows. The columns corresponding to the 2-OG binding residues (ALKBH5 Arg-277, and Arg-283) are indicated by purple arrows and the residues involved in 2-OG stabilization (ALKBH5 Asn-193, Tyr-195) are indicated by yellow arrows. The Gypsy_Pa_2799 sequence name is highlighted by a red box.

To address its putative function, we first compared the Gypsy-encoded ORF2 containing the 2-ODD domain to the NCBI protein database and noticed the highest similarity against plant proteins belonging to the alpha-ketoglutarate-dependent hydroxylase (AlkB) superfamily, which contains the 2-ODD fold. The TE-encoded 2-ODD domain was translated from consensus Gypsy_Pa_2799 and aligned with those from human and *Arabidopsis* reference proteins to determine its position in the AlkB superfamily using phylogenetic analysis. The resulting tree shows that the Gypsy-encoded AlkB domain is nested within *Arabidopsis* AlkBH5 homologs in a clade being sister to human AlkBH5 (Figure 4A). The 2-ODD-containing ORF2 from *Phalaenopsis aphrodite* Gypsy elements therefore represents a hypothetical AlkBH5 homolog and it is named paALKBH5-like hereafter. It is interesting to denote that, similar to hsALKBH5, some of the most closely related orthologs in *Arabidopsis*, atALKBH9B (AT2G17970) and atALKBH10B (AT4G02940), have been shown to bind RNA and to have m6A demethylase activity (Hu et al., 2019). We next addressed whether the sequences of TE-encoded proteins might conserve the amino acid residues necessary for this activity. The crystal structure of human AlkBH5 protein has revealed the residues binding the metal ion and 2-oxoglutarate (2-OG) cofactors and protein alignment indicated the strong conservation of these residues across the AlkB superfamily (Feng et al., 2014; Xu et al., 2014). Here, aligning the Gypsy-encoded 2-ODD domain with those of reference homologs revealed that all the cofactor-binding residues are conserved in the TE-encoded protein (Figure 4B). Thus, we observed that the known HxD/E⋅⋅⋅H and RxxxxxR motifs (indicated as red and purple arrows, respectively, in Figure 4B) involved in the binding to iron [Fe (II)] and 2-OG (Bratlie and Drablos, 2005; Lu et al., 2014) are present in the paALKBH5-like protein. As well, the N and Y residues involved in 2-OG stabilization are also conserved (indicated as yellow arrows in Figure 4B). Furthermore, the motif YNF potentially involved in the binding to m6A and an arginine residue essential for hsALKBH5 activity (Feng et al., 2014) are also conserved (residues are highlighted in fuchsia and green, respectively, on Supplementary Figure 2).

### Gypsy-Encoded 2-ODD Domain Has m6A Demethylase Activity Against ssRNA

**FIGURE 5:**
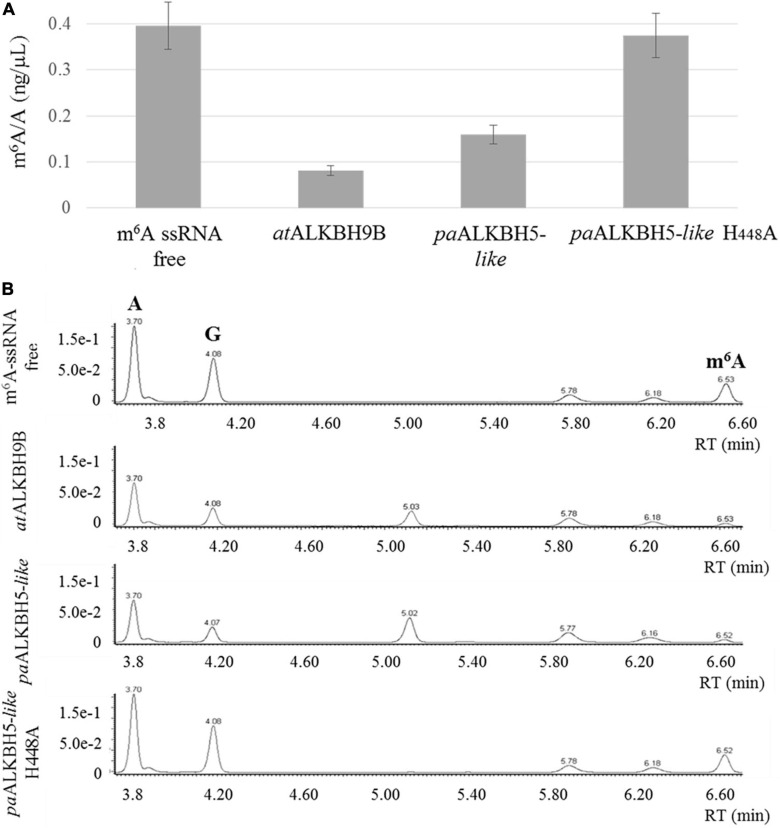
*In vitro* demethylation assay using ssRNA m^6^A-monomethylated. **(A)** Graphic showing the average m^6^A/A ratios obtained by UPLC-Q-TOF-MS after enzymatic digestion of the ssRNA m^6^A-monomethylated. The demethylation activity was evaluated in two independent experiments. **(B)** Representative UPLC-PDA-Q/TOF-MS chromatogram showing the retention times of the nucleosides adenosine “A” and N^6^-methyladenosine “m^6^A” after incubation of the m^6^A-containing ssRNA with non-protein (m^6^A-ssRNA free), *at*ALKBH9B, *pa*ALKBH5-*like* and *pa*ALKBH5-like H448A. The peak “G” corresponds to the nucleoside guanosine.

To assay the m6A demethylation activity of the *P. aphrodite* Gypsy-encoded 2-ODD domain, the paALKBH5-like protein was amplified and cloned in a bacterial expression plasmid fused to a Histidine tag at its C-terminal part. As positive control, *Arabidopsis* ALKBH9B protein was cloned and expressed in the same conditions. This protein was previously found to present m6A demethylation activity toward ssRNA (Martínez-Pérez et al., 2017). Thus, m6A monomethylated ssRNA were incubated with or without the recombinant proteins. Afterward, ssRNA was digested to single nucleoside and the resultant products were analyzed by ultra-performance liquid chromatography–photodiode detector–quadrupole/time-of-flight–mass spectrometry (UPLC-PDA-TOF-MS) as previously described (Martínez-Pérez et al., 2017). As shown in Figure 5, paALKBH5-like protein demethylated m6A on the ssRNA-oligonucleotide substrate by up to 60%, a percentage comparable to that observed for the positive control (ALKBH9B, 75%). Therefore, paALKBH5-like is a protein with ssRNA m6A demethylase activity “*in vitro.*” Previously it was found that change of the histidine at position 204 by alanine (H204A) in the characteristic iron ligand domain of the AlkB family (H204xD206…H266; Lu et al., 2014) of human ALKBH5 completely abolished the demethylation activity of the protein, confirming that the iron center was implicated in this activity (Zheng et al., 2013). The equivalent H204 is located at position 448 (His448, Asp450, and His496) in paALKBH5-like. To check if the putative iron center in paALKBH5-like was required for its demethylation activity, a recombinant version of the protein having the mutation H448A was generated and biochemically tested. We found that the m6A levels were similar to those of the negative control indicating that the change of H448 by A drastically affects the demethylase capacity of paALKBH5-like (Figure 5).

### Identification of a Putative RNA Binding Site

**FIGURE 6:**
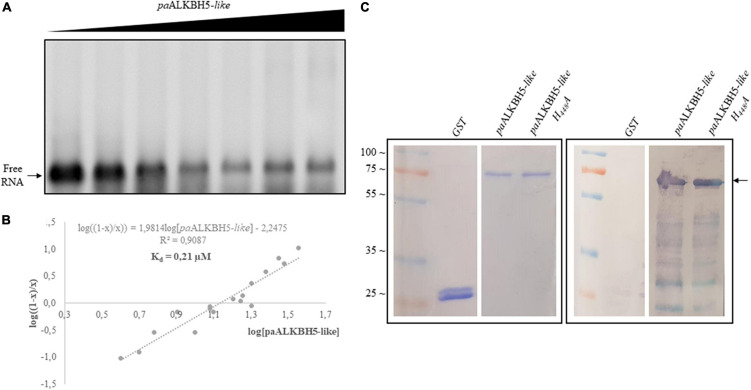
Analysis of the RNA binding capacity of paALKBH5-like. **(A)** EMSA after incubation of 5 ng of AMV 5′UTR transcript with no protein (lane 1) or with 5, 50, 100, 125, 175, 200, and 250 ng of His:paALKBH5-like protein. **(B)** Representation of Hill transformation of RNA-His:paALKBH5-like obtained from three independent experiments. R2 coefficient, regression equation and the dissociation constant (Kd) are shown. **(C)** Northwestern blot analysis result of the incubation of 1.5 μg GST, His:paALKBH5-like or its mutant variant H448A (indicated on the top) with 50 ng of AMV RNA 4 labeled with digoxigenin. The left panel shows the Coomassie blue-stained gel, and the right panel shows the nitrocellulose membrane after incubation. Positions of full-length His:paALKBH5-like proteins are indicated with an arrow. Ladder molecular mass is indicated on the left in kiloDaltons (KDa). Lines of the markers and GST were not contiguous to those of His:paALKBH5-like or its mutant variant H448A.

Recently, a biochemical analysis to delimit functional domains in *Arabidopsis* ALKBH9B showed two intrinsically disordered regions (IDRs) located at N- and C-terminal parts (Alvarado-Marchena et al., 2021). IDRs are unstructured regions implicated in protein–protein and RNA–protein interactions, which among others, present RGG boxes or R/K basic domains (repeats of arginine/glycine domains or arginine- or lysine-rich regions, respectively) [reviewed in Corley et al. (2020) and Ottoz and Berchowitz (2020)]. In ALKBH9B, an RNA binding domain containing an RGxxxRGG overlaps with the C-terminal IDR (Alvarado-Marchena et al., 2021). Both, C-terminal IDR and the RNA binding domain are absent in the TE-encoded protein (ORF2). However, we could detect an IDR in the N-terminal part of ORF2 and we noticed that it contains a basic domain consisting of a path rich with R and K residues (Supplementary Figure 2). Arginine residues have been found to interact with the phosphodiester backbone and in this sense RNA binding domain consisting of rich K and R patches have been identified in several RNA binding proteins (Ottoz and Berchowitz, 2020). Thus, is it tempting to speculate that this internal K/R rich domain would confer RNA binding capabilities to the Gypsy_Pa_2799 protein. To check this possibility we carried out Electrophoretic Mobility Shift Assays (EMSA) by incubating a constant amount (5 ng) of an RNA transcript, corresponding to the 5′ untranslated region (UTR) of AMV RNA3, with increasing concentrations of the paALKBH5-like (Gypsy_Pa_2799) protein (Figure 6A). The decrease in the chemiluminescent signal intensity corresponding to free RNA was evident at quantities exceeding 125 ng of the protein. The apparent constant dissociation (Kd) of the RNA–paALKBH5-like interaction from the linear regression of the mean values from at least three technical replicates (Marcos et al., 1999) was estimated to be 0.21 μM (Figure 6B). To determine if the mutant lacking demethylase activity retained its RNA-binding capacity, Northwestern experiments were performed with both proteins. As can be seen in Figure 6C, the His:paALKBH5-like H448A mutant in which its demethylase activity was abolished maintained its RNA binding capacity, indicating that this capacity is necessary but not sufficient for its function.

## Discussion

In this study, we screened the content of conserved protein domains in LTR retrotransposon consensus sequences generated from a selection of plant genome assemblies. Admittedly, the set of domains found is biased by the approach used to build consensus sequences, which is empirically not adapted to the analysis of low-copy number TE families and which efficiency decreases with low contiguity genome assemblies. This analysis is also biased by the selection of the species investigated here. Nevertheless, we generated thousands of consensus sequences representative of Copia-type and Gypsy-type LTR retrotransposons and the analysis of their protein domain content allowed to successfully retrieve several domains that were previously reported in plant Gypsy-type elements such as CHROMO, PMD, and AIR1. In line with the literature, no domain gain was observed in plant Copia-type elements by contrast to plant Gypsy elements which are more prone to domain acquisition and/or retention.

We noticed that the most frequent protein domains described have functional categories relevant to interactions with RNA and DNA including AlkB, G-patch, KOW, ArgoL2, B3, and potentially AIR1 domain, which overlaps UMSBP DNA binding domain. Given that TEs have compact genomes with limited coding capacity, the retention of these domains through evolution and natural selection suggests that they bring important functions to the cognate TE families that result in a positive impact on TE fitness (with an acceptable impact on host fitness). Consequently, such a functional bias suggests that these fixed domains may have been selected out of a cryptic flow of domain acquisition events, the majority of which being rejected throughout evolution. Remarkably, several of these domains have been acquired independently in different mobile genetic elements. For instance, PMD domain is also found in MULE DNA transposons (Babu et al., 2006). The G-patch has also been detected in type D retroviruses (Aravind and Koonin, 1999). Furthermore, AlkB domain was reported in several families of RNA viruses, including Alphaflexiviridae, Betaflexiviridae, and Closteroviridae (Aravind and Koonin, 2001; Moore and Meng, 2019). The virus-encoded proteins display RNA demethylation activity *in vitro* and it has been postulated that its RNA repair function could help to maintain genome integrity (van den Born et al., 2008).

Here, we described that a specific family of Phalaenopsis Gypsy-type elements has acquired an additional ORF and retained it over several rounds of replication as attested by the diversity of consensus sequences reconstructed for this family (Figure 2A) which probably reflects speciation into different subfamilies after the ORF was captured. This ORF contains a 2-ODD domain, conserves the cofactor-binding residues necessary for its activity (Figure 4B), is most closely related to known RNA demethylases from human and *Arabidopsis* (Figure 4A) and, most importantly, the purified protein shows RNA demethylase activity *in vitro* (Figure 5) and capability to bind RNA (Figure 6). Interestingly, the apparent constant dissociation (Kd) calculated for the paALKBH5-like protein (0.21 μM, Figure 6) was similar to that observed for atALKBH9B (0.30 μM) (Alvarado-Marchena et al., 2021) reinforcing the notion that both proteins are functionally equivalent.

Following the “no superflue” postulate regarding the genomes of functional and autonomous TE sequences, one can suppose that its demethylase activity could bring selective advantage to the TE family itself, e.g., by increasing its fitness. After transcription, the LTR retrotransposon mRNA is exported to the cytoplasm where it is translated (Sabot and Schulman, 2006). We can speculate that, upon translation, the RNA demethylase protein (ORF2), which is expressed independently of the Gag-Pol polyprotein (ORF1), could bind and be enzymatically active against the cognate TE mRNA or any cellular RNA. To bring any advantage to the TE, this could suppose either that the TE mRNA is subject to m6A methylation by the host and that it is deleterious to the TE replication cycle or/and that the demethylation of other cellular RNA by TE-encoded AlkB protein impacts the stability and translation of genes involved in different layers of TE control, as recently proposed for some AlkB-encoding plant RNA viruses (Yue et al., 2022).

m6A methylated mRNAs recruit specific reader proteins and, in mammals, it has been found that a polymethylated mRNA recruits several reader molecules causing the juxtaposition of their intrinsically disordered regions (IDRs) that activates the phenomenon of liquid-liquid phase separation. Thus, the polymethylated mRNA-eraser protein complexes accumulate into different phase-separated membraneless compartments such as stress granules or P- bodies that affect either the stability or translation of these mRNAs (Ries et al., 2019). Plant m6A readers also contain IDRs and some of them have been found to form dense assemblages *in vitro* and accumulate in granules upon stress (Arribas-Hernandez et al., 2018). Thus, the abundance of m6A sites in TE-derived mRNAs might influence their expression and retrotransposition activities.

Most recently, it has been reported in human cells that RNA transcripts of young LINE-1 retrotransposons present elevated levels of m6A modification that acts promoting their expression and retrotransposition (Xiong et al., 2021). In *Arabidopsis*, most TE transcripts exhibit a relatively high extent of m^6^A modification compared to gene transcript level (Wan et al., 2015). However, this has been observed under standard conditions in which mostly fragmented forms of TE transcripts can be detected. It would be interesting to measure the level of m6A methylation in TE mRNA in the context of their replicative cycles and to address its impact on the regulation of the transposition rates. Remarkably, a recent report deposited in bioRxiv shows that the RNA of the heat-activated retroelement ONSEN contains m6A, which suppresses the transposon mobility via its sequestration in stress granules in an m6A-dependent manner. Furthermore, the RNA demethylase AtALKBH9B directly demethylates ONSEN m6A-modified RNA located in stress granules allowing its mobilization. In fact, it has been proposed that in plants, some RNA demethylases would have diversified to preferentially target genetic elements such as retrotransposons (Fan et al., 2022).

## Data Availability Statement

The datasets presented in this study can be found in online repositories. The names of the repository/repositories and accession number(s) can be found in the article/Figshare: https://figshare.com/projects/Orchid_Ogre_with_RNA_demethylase_-_Supplementary_dataset/137215.

## Author Contributions

LA-M and MM-P performed the experiments. VP supervised the experiments. FM performed the bioinformatic analyses and conceived the study. FM, FA, MM-P, and VP interpreted the results and wrote the manuscript. All authors analyzed and discussed the results.

## Conflict of Interest

The authors declare that the research was conducted in the absence of any commercial or financial relationships that could be construed as a potential conflict of interest.

## Publisher’s Note

All claims expressed in this article are solely those of the authors and do not necessarily represent those of their affiliated organizations, or those of the publisher, the editors and the reviewers. Any product that may be evaluated in this article, or claim that may be made by its manufacturer, is not guaranteed or endorsed by the publisher.
